# Identification, Characteristics and Mechanism of 1-Deoxy-*N*-acetylglucosamine from Deep-Sea *Virgibacillus dokdonensis* MCCC 1A00493

**DOI:** 10.3390/md16020052

**Published:** 2018-02-07

**Authors:** Dian Huang, Zong-Ze Shao, Yi Yu, Min-Min Cai, Long-Yu Zheng, Guang-Yu Li, Zi-Niu Yu, Xian-Feng Yi, Ji-Bin Zhang, Fu-Hua Hao

**Affiliations:** 1 State Key Laboratory of Agricultural Microbiology, National Engineering Research Center of Microbe Pesticides, College of Life Science and Technology, Huazhong Agricultural University, Wuhan 430070, China; hui8229hd@163.com (D.H.); cmm114@mail.hzau.edu.cn (M.-M.C); ly.zheng@mail.hzau.edu.cn (L.-Y.Z.); yz41@mail.hzau.edu.cn (Z.-N.Y.); 2 Key Laboratory of Marine Biogenetic Resources, Third Institute of Oceanography, State Oceanic Administration, Xiamen 361005, China; shaozz@163.com (Z.-Z.S.); mccc_ligy@163.com (G.-Y.L.); 3 School of Pharmaceutical Sciences, Wuhan University, Wuhan 430070, China; yuyi119@hotmail.com; 4 Key Laboratory of Magnetic Resonance in Biological Systems, State Key Laboratory of Magnetic Resonance and Atomic and Molecular Physics, Wuhan Centre for Magnetic Resonance, Wuhan Institute of Physics and Mathematics, Chinese Academy of Sciences, Wuhan 430071, China; yxf@wipm.ac.cn

**Keywords:** *Virgibacillus dokdonensis*, *Xanthomonas oryzae* pv. *oryzae*, 1-Deoxy-*N*-acetylglucosamine, antibacterial characteristics, mechanism

## Abstract

*Xanthomonas oryzae* pv. *oryzae*, which causes rice bacterial blight, is one of the most destructive pathogenic bacteria. Biological control against plant pathogens has recently received increasing interest. 1-Deoxy-*N*-acetylglucosamine (1-DGlcNAc) was extracted from the supernatant of *Virgibacillus dokdonensis* MCCC 1A00493 fermentation through antibacterial bioassay-guided isolation. Its structure was elucidated by LC/MS, NMR, chemical synthesis and time-dependent density functional theory (TD-DFT) calculations. 1-DGlcNAc specifically suppressed *X. oryzae* pv. *oryzae* PXO99A (MIC was 23.90 μg/mL), but not other common pathogens including *Xanthomonas campestris* pv. *campestris* str.8004 and *Xanthomonas oryzae* pv. *oryzicola* RS105. However, its diastereomer (2-acetamido-1,5-anhydro-2-deoxy-d-mannitol) also has no activity to *X. oryzae* pv. *oryzae.* This result suggested that activity of 1-DGlcNAc was related to the difference in the spatial conformation of the 2-acetamido moiety, which might be attributed to their different interactions with a receptor. Eighty-four unique proteins were found in *X. oryzae* pv. *oryzae* PXO99A compared with the genome of strains8004 and RS105 by blastp. There may be unique interactions between 1-DGlcNAc and one or more of these unique proteins in *X. oryzae* pv. *oryzae*. Quantitative real-time PCR and the pharmMapper server indicated that proteins involved in cell division could be the targets in PXO99A. This research suggested that specificity of active substance was based on the active group and spatial conformation selection, and these unique proteins could help to reveal the specific mechanism of action of 1-DGlcNAc against PXO99A.

## 1. Introduction

*Xanthomonas oryzae* pv. *oryzae*, which causes rice bacterial blight, is an important pathogen in rice. The disease was first observed by Japanese farmers in 1884 [1] and causes severe economic losses in agriculture worldwide [2,3]. The whole-genome sequences of three *X. oryzae* pv. *oryzae* strains (KACC10331, MAFF311018, and PXO99A) have been reported [4,5,6], and a series of genes and regulatory proteins associated with pathogenesis have been found in *X. oryzae* pv. *oryzae* [7,8,9]. In addition, many blight resistance genes also have been identified in rice [10], and the unique rice-*X. oryzae* pv. *oryzae* pathosystem has also been revealed [11]. Biological control of *X. oryzae* pv. *oryzae* by terrestrial microorganisms has been widely reported [12,13,14].

The marine environment is recognized as a rich source of metabolites with diverse biological activities [15,16,17]. More than 22,000 secondary metabolites have been isolated from marine microorganisms, and have been structurally characterized and evaluated for biological activities such as antimicrobial or antitumor activities. However, the compounds identified in many exciting discoveries still need a broader range of evaluation [18].

The genus *Virgibacillus*, which was originally designated as *Bacillus* spp., was first reported by Heyndrickx et al. [19]. A few *Virgibacillus* strains were found to have antifungal behavior. For example, Essghaier et al. [20] reported that a moderately halophilic bacterium from a Tunisia salt lake (*V. marismortui* M3-23) has antimicrobial activity against the phytopathogenic fungus *Botrytis cinerea* through the functions of extracellular proteins such as chitinase and glucanase. However, no active compound against phytopathogenic bacteria has been found in the genus *Virgibacillus*. We hypothesized that the bacterium may produce different types of bioactive secondary metabolites inhibiting various pathogens.

In this study, *V. dokdonensis* MCCC 1A00493 was isolated from deep-sea polymetallic nodules in the East Pacific Ocean. The objectives of this study were to isolate the bioactive secondary metabolites from *V. dokdonensis* MCCC 1A00493 under the guidance of bioassays, to determine their chemical structures, and to evaluate their antimicrobial characteristics.

## 2. Results and Discussion

### 2.1. Structure Identification of the Antibacterial Substance

**Figure 1:**
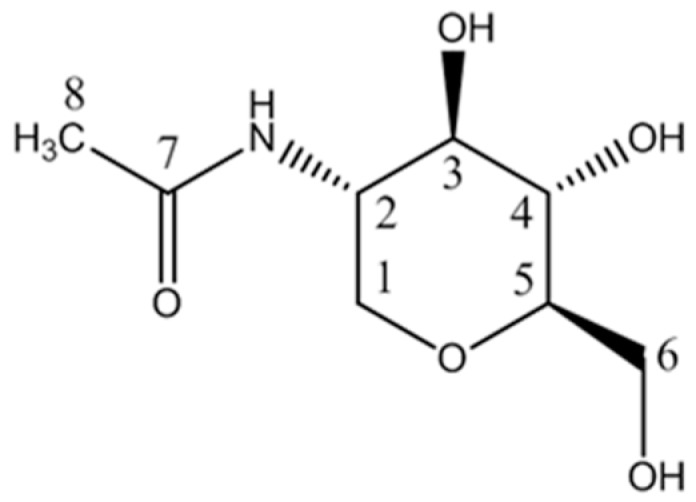
The planar structure of 1-DGlcNAc.

The isolated antibacterial substance (18.3 mg) was obtained as a white powder soluble in methanol and H_2_O. It showed an [M + H]^+^ ion at *m/z* 206.1022 and a [2M + Na]^+^ ion at *m/z* 433.1794 in the LC-ESI-MS analysis, indicating a molecular weight of 205, compatible with the molecular formula C_8_H_15_NO_5_. The IR spectrum displayed absorption bands typical of hydroxyl (3435.27) and carbonyl groups (1653.29). The specific rotation ([α ${]}_{D}^{20.0}$) value was +18.5 (*c* 0.1, H_2_O). The ring structure of 1-DGlcNAc was confirmed through the ^1^H spectrum (Figure S1) and a series of 2D NMR spectra (Figures S2−S6). The ^1^H and ^13^C NMR spectroscopic signals were suggestive of an analogue of 2-acetamido-1,5-anhydro-2-deoxy-d-mannitol [21]. One ^1^H−^1^H coupling system was observed in the TOCSY spectrum (Figure S2), suggesting the eight correlated protons at *δ*_H_ 3.90, 3.84, 3.81, 3.63, 3.41, 3,28, 3.19 and 3.13, which was confirmed with data from COSY (Figure S3), JRES (Figure S4), HSQC (Figure S5) and HMBC spectra (Figure S6). Sequential positions of these protons were determined as *δ*_H_ 3.90/3.13 − 3.81 − 3.41 − 3.28 − 3.19 − 3.84/3.63 with the COSY spectrum. In the HSQC spectrum, the direct H−C correlations were determined; furthermore, two pairs of signals for protons at *δ*_H_ 3.90 and 3.13 ppm, and 3.84 and 3.63 ppm, which correlated in the HSQC spectrum with carbon signals at *δ*_C_ 69.2 and 63.2 ppm, respectively, indicated the presence of two oxygenated methylenes in the molecule. Through the HMBC spectrum, the strong long-range correlation for CH_3_ (*δ*_H_ 1.97)/*δ*_C_ 174.3 suggested the presence of the CH_3_−CO- moiety, and weak long-range correlations for CH_3_/C1, C2, C3 (*δ*_H_ 1.97/*δ*_C_ 69.2, 53.2, 77.0), combined with IR data, together indicated the presence of the CH_3_−CO-NH–C2- moiety. In addition, long-range correlations for H1, H1’/C5 (*δ*_H_ 3.13, 3.90/*δ*_C_ 82.7,) and H5/C1 (*δ*_H_ 3.19/*δ*_C_ 69.2,) suggested a C1-O-C5 moiety. Therefore, the planar structure of 1-DGlcNAc was determined as shown in Figure 1. The detailed assignments of ^1^H and ^13^C signals are showed in Table 1.

In order to confirm the absolute configuration of 1-DGlcNAc, we further calculated the J-coupling constants of all sixteen possible isomers for this compound with density functional theory (DFT) calculations using the GIAO method at the ωB97XD/6-31G(d,p) level following full optimization for all the structures at the same level with Gaussian 09. By comparing the calculated values with experimental coupling constants, the relative configuration (2*S*, 3*R*, 4*S*, 5*R*) was proposed (data not shown). Furthermore, the experimental ECD data of this compound were also in good agreement with the calculated result (Figure S8). Thus, the absolute configuration of this compound was established as 2-acetamido-1,5-anhydro-2-deoxy-d-glucitol, also known as 1-DGlcNAc.

The synthesized sample from WuXi AppTec also validated the results of the NMR analysis. The chemical synthesis has characteristics of simple synthesis, cheap raw material and stable product. By comparing the NMR and ECD data of the isolated natural compound with those of the chemically synthesized compound, we found that the isolated compound shared the same chemical shift and ECD result with 1-DGlcNAc (Figures S7 and S8).

1-DGlcNAc was first found as a product of a chemical reaction in 1956 [22], and this is the first report of its isolation and purification from a natural source, and its biological activity.

### 2.2. Antimicrobial Bioassay

The antimicrobial spectrum of 1-DGlcNAc is shown in Table 2. The compound displayed strong inhibitory activity against *X. oryzae* pv. *oryzae*, with a MIC value of 23.90 μg/mL. The deletion of *PXO_00069* in PXO99A attenuated extracellular polymeric substance (EPS) synthesis and swarming ability [23]. The mutant does not produce xanthan gum, but it was still sensitive to the compound. 1-DGlcNAc had no antimicrobial activity against other pathogens tested. The related compound 2-acetamido-1,5-anhydro-2-deoxy-d-mannitol showed no antimicrobial activity against 10 fungi and 9 bacteria. This compound has been reported as a potential inhibitor of sialic acid biosynthesis [21], while 1-DGlcNAc can inhibit the feeding behavior of rats [24,25], but there has been no prior report about its antimicrobial activity. Here we reported that 1-DGlcNAc could specifically and efficiently suppress *X. oryzae* pv. *oryzae.* By blastp, the proteins among *X. oryzae* pv. *oryzae* PXO99A, *X. campestris* pv. *campestris* str.8004 and *X. oryzae* pv. *oryzicola* RS105 were compared. Eighty-four unique proteins were found in *X. oryzae* pv. *oryzae* PXO99A (data not shown). There may be unique interactions between 1-DGlcNAc and one or more of these unique proteins in *X. oryzae* pv. *oryzae*. In our future study, we will determine the mechanism involved in 1-DGlcNAc mediated protection against *X. oryzae* pv. *oryzae* in rice.

Chiral molecules are of great significance in life science [26]. In the world of life, proteins, nucleic acids, hormones, phospholipids, saccharides, sterols, pheromones and so on are all chiral [27]. It has been well established that epimers of a given compound can have distinct biological properties. 2-acetamido-1,5-anhydro-2-deoxy-d-glucitol and its diastereomer 2-acetamido-1,5-anhydro-2-deoxy-d-mannitol also show different bioactivities to *X. oryzae* pv. *oryzae.* The only structural difference between them is the spatial conformation of the 2-acetamido moiety, which might account for different interactions with a receptor, and could help to reveal the mechanism of action of 1-DGlcNAc against PXO99A.

### 2.3. Analysis of Xanthomonas Potential Target Gene Expression after Exposure to 1-DGlcNAc

**Figure 2:**
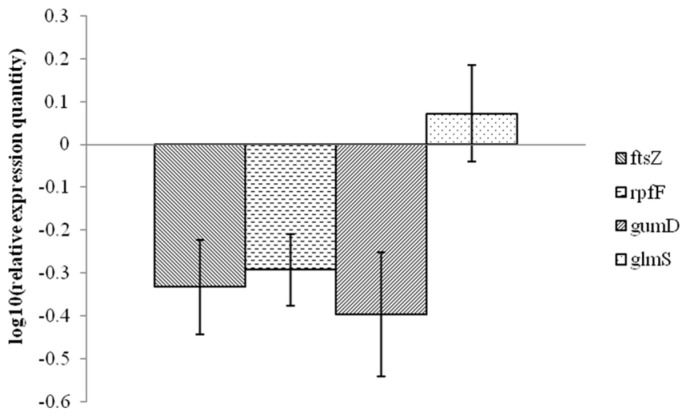
Quantitative real-time PCR analysis of expression of four genes (*rpfF*, *gumD*, *ftsZ*, *glmS*) in *X. oryzae* pv. *oryzae* PXO99A in response to 1-DGlcNAc treatment. Values were normalized to the levels of *16S rRNA*, an internal reference gene. The y-axis represents mean expression values ± SD relative to the control. The experiment was independently repeated three times.

Four genes (*rpfF*, *gumD*, *ftsZ* and *glmS* in *Xanthomonas* spp.) were chosen to explore the effects of 1-DGlcNAc on *Xanthomonas* gene expression. The *rpfF* gene is involved in production of a diffusible signal factor (DSF) [28] and the *gumD* gene is part of the *gum* operon responsible for extracellular polysaccharide (EPS) biosynthesis [29], which are both required for virulence. The *ftsZ* gene product is involved in cell division [30]. The *glmS* gene encodes glucosamine-6-phosphate synthase, which is important for the biosynthesis of peptidoglycan, a component of the bacterial cell wall [31]. Figure 2 shows that the transcriptional expression of *rpfF*, *gumD* and *ftsZ* was downregulated, while the levels of *glmS* were barely changed upon treatment of *X. oryzae* pv. *oryzae* with 1-DGlcNAc. The expression of *glmS* could be linked to the response to xenobiotics. The downregulated *ftsZ* gene resulted in fewer pathogenic bacteria. The transcript levels of virulence genes *rpfF* and *gumD* decreased, which coincided with a decline in disease severities.

### 2.4. Morphological and Ultrastructural Changes of Xanthomonas Cells in the Presence of 1-DGlcNAc 

**Figure 3:**
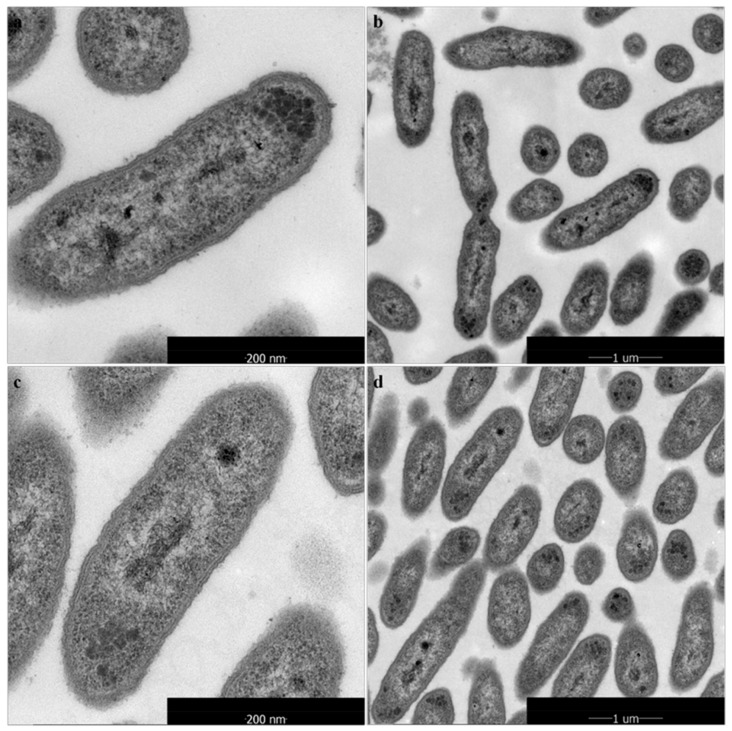
Ultrastructural effects of 100 μg/mL 1-DGlcNAc on *Xanthomonas* cells after 3 h determined by TEM. (**a**,**b**) untreated *X. oryzae* pv. *oryzae* cells; (**c**,**d**) treated *X. oryzae* pv. *oryzae* cells.

**Figure 4:**
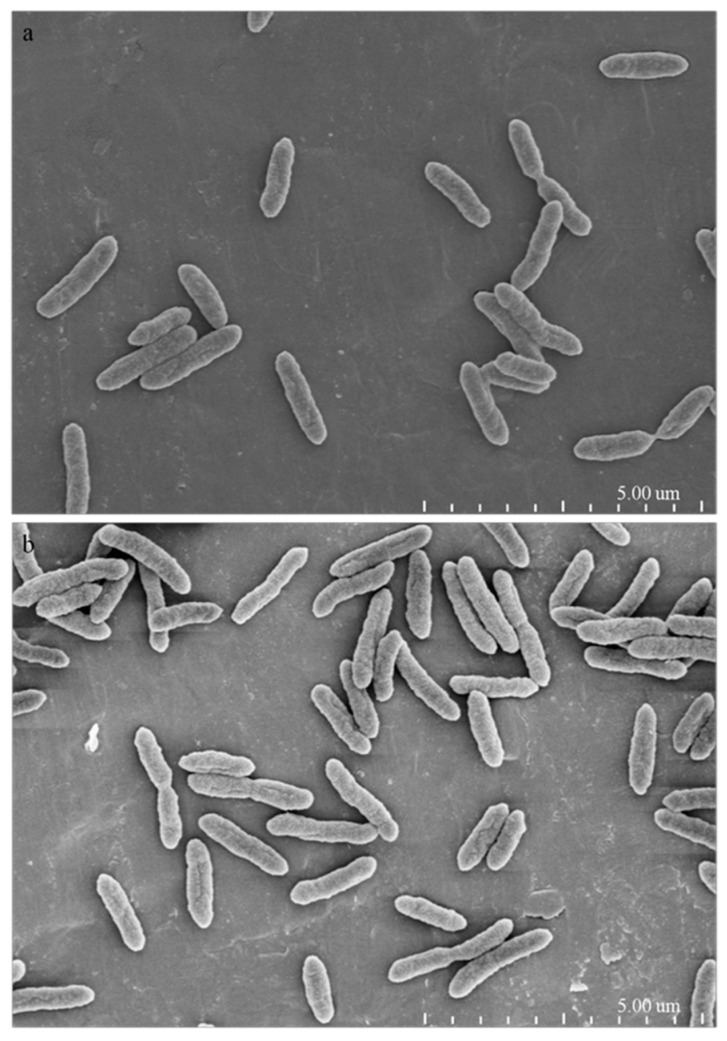
Ultrastructural effects of 100 μg/mL 1-DGlcNAc on *Xanthomonas* cells after 3 h determined by SEM. (**a**) untreated *X. oryzae* pv. *oryzae* cells; (**b**) treated *X. oryzae* pv. *oryzae* cells.

Visualization at the ultrastructural level of the cellular damage of *X. oryzae* pv. *oryzae* caused by 1-DGlcNAc was undertaken by SEM and TEM analyses. In the SEM study, untreated control cells appeared intact, plump and typically rod-shaped with a smooth exterior (Figure 3a,b). Upon exposure to 1-DGlcNAc, cell walls became a little tighter (Figure 3c,d), consistent with the qRT-PCR result that glmS expression increased slightly. By TEM, untreated cells showed a very distinct cell wall and a uniformly distributed electro-dense cytoplasm (Figure 4a). After 3 h of treatment with 1-DGlcNAc, there was no evident lysis of the bacterial cell or efflux of intracellular components. Treated cells showed a similar morphological structure with the untreated cells (Figure 4b). According to the morphological and ultrastructural changes, 1-DGlcNAc had no significant influence on the *Xanthomonas* cell structure.

### 2.5. Prediction of 1-DGlcNAc Potential Target Proteins

The top pharmacophore candidates identified via pharmMapper are listed in Table S2, and the potential target proteins are shown in Table 3. The ranked list of hit target pharmacophore models is sorted by normalized fit score in descending order. Four pharmacophores among the top 300 candidates are annotated as potential targets, namely cell division protein kinase 2 (ranked 2), cell division control protein 2 homolog (ranked 64), cell division control protein 42 homolog (ranked 235) and cell division protein ftsZ (ranked 239). We inferred that 1-DGlcNAc could target the proteins involved in *X. oryzae* pv. *oryzae* cell division, block cell division and cause cell death. 

Xanthan, a characteristic EPS produced by *X. oryzae* pv. *oryzae*, can block the water flow in rice xylem vessels and provide an advantage for *X. oryzae* pv. *oryzae* against environmental stresses [32]. Xanthan gum is a complex exopolysaccharide assembled stepwise from UDP-glucose, GDP-mannose, and UDP-glucuronic acid. Therefore, the synthesis of xanthan would be impacted by the galactose metabolic pathway. *X. oryzae* pv. *oryzae* PXO99Δ00069 does not produce xanthan gum, but it was still sensitive to the compound 1-DGlcNAc, demonstrating that xanthan gum was not a possible factor in the activity of the inhibitor.

As the world population grows, rice demand is expected to increase by at least 25% by 2030 [33]. This study demonstrated the antibacterial characteristics of 1-DGlcNAc and suggested that specificity of the active substance was based on the active group and spatial conformation selection, and one or more of these unique proteins in *X. oryzae* pv. *oryzae* could help to reveal the specific mechanism of action of 1-DGlcNAc against PXO99A. 

## 3. Experimental Section

### 3.1. Pathogens and Chemicals

The pathogen *X. oryzae* pv. *oryzae* wild type PXO99A, mutant PXOΔ00069, and *X. campestris* pv. *campestris* str.8004 were provided by the State Key Laboratory of Agricultural Microbiology, Huazhong Agricultural University, China. *X. oryzae* pv. *oryzicola* RS105 was provided by the Hubei Academy of Agricultural Science. *Escherichia coli*, *Staphylococcus aureus*, *Salmonella typhimurium*, *Pseudomonas solanacearum*, *Pseudomonas syringae*, *Rhizoctonia solani* Kühn, *Aspergillus niger*, *Penicillium italicum* Wehmer, *Fusarium oxysporum* f. sp. *Cubense*, *Fusarium graminearum* Sehw, *Fusarium oxysporium* f. sp. *vasinfectum*, *Magnaporthe oryzae*, *Botrytis cinerea* Pers, *Rhizoctonia solani* Kühn, and *Sclerotinia sclerotiorum* (Lib.) de Bary were provided by the Culture Center of Agricultural Microbes, Huazhong Agricultural University, China. 

1-DGlcNAc was initially isolated from *V. dokdonensis* 1A00493 and subsequently synthesized (95%, WuXi AppTec Biopharmaceuticals, Tianjin, China) along with 2-acetamido-1,5-anhydro-2-deoxy-d-mannitol (95%) for the in vitro studies.

### 3.2. Strain isolation and Fermentation

411 marine isolates from the Marine Culture Collection Center of China (MCCC) were screened for activity in vitro against bacterial and fungal plant pathogens. Finally, strain *V. dokdonensis* 1A00493, which was originally isolated from deep-sea polymetallic nodules in the East Pacific Ocean, was selected for further analysis.

Strain 1A00493 was cultivated for 12 h in a 250-mL Erlenmeyer flask containing 50 mL modified marine bacterial medium 2216e (tryptone 10 g, yeast extract 5 g, beef extract 1 g, CH_3_COONa·3H_2_O 1 g, NH_4_NO_3_ 0.2 g, C_6_H_5_Na_3_O_7_·2H_2_O 0.5 g, C_6_H_5_O_7_Fe·xH_2_O 0.1 g, artificial seawater fixed to 1 L, pH 7.6 ± 0.2. artificial seawater formula: NaCl 19.45 g, MgCl_2_·6H_2_O 0.75 g, MgSO_4_·7H_2_O 0.75 g, CaCl_2_ 1 g, KCl 0.55 g, NaHCO_3_ 0.16 g, KBr 0.08 g, SrCl_2_·6H_2_O 34 mg, H_3_BO_3_ 22 mg, Na_2_SiO_3_·9H_2_O 4 mg, NaF 2.4 mg, Na_2_HPO_4_·12H_2_O 8 mg, MnCl_2_·4H_2_O 0.5 mg, CuSO_4_·5H_2_O 0.5 mg, ZnSO_4_·7H_2_O 10 mg, add ddH_2_O to 1 L). The seed culture was then transferred to 200 mL of modified 2216e medium in 500-mL Erlenmeyer flasks and incubated at 28 °C for 48 h with shaking at 180 revolutions/min.

### 3.3. Extraction and Isolation of the Active Substance 

The fermented culture (10 L) was centrifuged and the supernatant was collected. The supernatant was extracted with an equal volume of MeOH at 4 °C for 24 h to denature protein and polymers, the mixture was centrifuged, and the supernatant was evaporated under reduced pressure to obtain the crude extract. This extract was chromatographed on a silica gel column using a stepwise gradient elution of petroleum ether, petroleum ether: EtOAc (1:1), EtOAc: MeOH (1:1) and MeOH. The EtOAc:MeOH eluent, which was found to contain the active substance by ninhydrin reaction analysis, was further fractionated on a reverse-phase silica gel column with a gradient elution of acetonitrile in H_2_O (10% to 15%). The fractions containing the active substance were combined, evaporated under reduced pressure, and subjected to Sephadex LH-20 column chromatography with 50% MeOH in H_2_O. The active fractions were combined, evaporated to dryness, dissolved in H_2_O and applied to a column (Phenomenex Luna 5µ NH_2_ 100A, 250 × 4.60 mm) for further purification by high performance liquid chromatography (HPLC: Shimadzu LC-20AT, Kyoto, Japan).Elution was with 80% acetonitrile in H_2_O and detection was with a differential refraction detector (RID-10A, Shimadzu, Kyoto, Japan). Isolation of the antibacterial substance (18.3 mg) was confirmed by in vitro antibacterial activity.

### 3.4. Spectroscopic and NMR Analysis 

Electrospray ionization-mass spectrometry (ESI-MS) spectral data were acquired with an Agilent 6540 UHD Accurate-Mass Q-TOF LS/MS spectrometer (Agilent Technologies, Singapore). Chromatographic analysis was achieved on a C18 column (particle size 5 mm, 100 × 2.1 mm, Agilent Technologies, Santa Clara, CA, USA). The mobile phase was 5% acetonitrile in H_2_O for 2 min followed by a gradient of acetonitrile (5–90%) for 10 min at a flow rate of 0.3 mL/min. IR spectra were measured on a Thermo Avatar 330 FT-IR spectrophotometer (Thermo Nicolet, Madison, WI, USA). Optical rotations were measured with a WZZ-2S Automatic Polarimeter (Shanghai Precision Instrument Co., Ltd., Shanghai, China). CD spectra were measured at room temperature on a Chirascan Circular Dichroism Spectrometer (Applied Photophysics Ltd., Leatherhead, UK).

For NMR analysis, about 10 mg of purified antibacterial sample was dissolved in 500 μL deuterated methanol (MeOD) and transferred into a 5 mm NMR tube. Data were acquired on a Bruker Avance III 600 MHz spectrometer (Bruker Biospin, Rheinstetten, Germany) equipped with a 5 mm cryogenic TCI probe at 298 K. Topspin software packages (Bruker, Rheinstetten, Germany) were used for NMR data acquisition and processing. The one-dimensional ^1^H NMR spectrum was recorded using noesygppr1d pulse sequence with 32 scans, 32 K data points, spectra width of 20 ppm, and recycle delay of 2 s. A series of 2D NMR spectra, including ^1^H−^1^H correlation spectroscopy (COSY), ^1^H−^1^H total correlation spectroscopy (TOCSY), ^1^H−^1^H J-resolved spectroscopy (JRES), ^1^H−^13^C heteronuclear single quantum coherence spectroscopy (HSQC) and ^1^H−^13^C heteronuclear multiple bond correlation spectroscopy (HMBC) were recorded and processed in a similar fashion as reported previously [34] with slightly different parameters. ^1^H and ^13^C chemical shifts were referenced to residual solvent signals of MeOD (*δ*^1^H = 3.31 and *δ*^13^C = 49.5).

### 3.5. Theoretical Calculation

The ^1^H−^1^H spin-spin coupling constants of sixteen configurations, according to the four chiral carbons of 1-DGlcNAc, were calculated based on the density functional theory (DFT). Prior to coupling calculations, all of the molecular geometries were fully optimized at the ωB97XD/6-31G(d,p) level of theory. Based on the optimized structures, the coupling constants were then calculated at the theoretical level. The calculated coupling constants of all configurations were subsequently compared with the experimental data to find the best possible configuration (2*S*, 3*R*, 4*S*, 5*R*). The theoretical calculation of ECD was conducted in H_2_O using TD-DFT at the ωB97XD/6-31G(d,p) level as well. Rotatory strengths for a total of 50 excited states were calculated. The ECD spectrum is simulated in SpecDis [35] by Gaussian functions. The calculated ECD spectra of (2*S*, 3*R*, 4*S*, 5*R*) of 1-DGlcNAc were further compared with the experimental spectrum. All calculations were carried out using the Gaussian 09 software package (Gaussian, Inc., Wallingford, CT, USA).

### 3.6. Chemical Synthesis of 1-DGlcNAc and Its Diastereomer 2-Acetamido-1,5-anhydro-2-deoxy-d-mannitol

**Figure 5:**
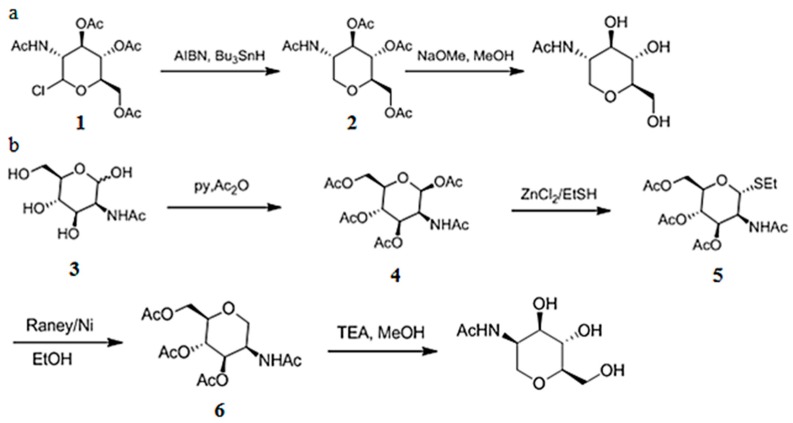
The synthesis procedures of 1-DGlcNAc (**a**), 2-acetamido-1,5-anhydro-2-deoxy-d-mannitol (**b**).

1-DGlcNAc and its diastereomer (2-acetamido-1,5-anhydro-2-deoxy-d-mannitol) were synthesized by WuXi AppTec (Tianjin, China). 1-DGlcNAc was synthesized as shown in Figure 5a. A solution of compound **1** (15.0 g, 41.0 mmol) in toluene (200 mL) was purged with N_2_ for 30 min, then Bu_3_SnH (14.3 g, 49.2 mmol) and AIBN (1.35 g, 8.20 mmol) was added and the mixture was stirred at 110 °C for 2 h. Compound **2** (9.60 g, 70% yield) was obtained as a white solid. Compound **2** (3.00 g, 9.05 mmol) in MeOH (30 mL) was added to NaOMe (977 mg, 18.1 mmol). The mixture was stirred at 25 °C for 1 h. Compound 1-DGlcNAc (1.02 g, 54% yield) was obtained as a white solid.

Its diastereomer was synthesized as shown in Figure 5b. Compound **3** (5.0 g, 23 mmol) in pyridine (50 mL) was mixed with Ac_2_O (12 g, 113 mmol) at 0–25 °C for 12 h. Compound **4** (8 g, 91% yield) was obtained as a white solid. Compound **4** (1.2 g, 3.08 mmol) in DCM (5 mL) was added to ZnCl_2_ (1 M, 4.62 mL) and EtSH (12 mL) at 0 °C, the mixture solution was stirred at 0 °C for 12 h, then heated to 40 °C for 3 h. Compound **5** (1.80 g, 50% yield) was obtained as a white foam solid. Raney-Ni (3.40 g, 39.7 mmol) was suspended in anhydrous ethanol (15 mL), stirred for 3 min, then let stand. EtOH was decanted. The process was repeated four times until the ethanol phase became clear after standing. 10 mL EtOH was added and then compound **5** (300.00 mg, 766.40 μmol) was added. Under stirring, the residue was de-gassed by bubbling N_2_ for 10 min, the mixture solution was heat under reflux a N_2_ atmosphere for 0.5 h. Compound **6** (120 mg, 16% yield) was obtained as a white solid. Compound **6** (120 mg, 362 μmol) in MeOH (1.00 mL) was added to NaOMe (9.9 mg, 181 μmol) at 25 °C for 1 h. 2-acetamido-1,5-anhydro-2-deoxy-d-mannitol (48.00 mg, 65% yield) was obtained as a white solid. The purity and structure of the products were checked by MS and NMR.

### 3.7. In Vitro Antibacterial and Antifungal Activity of 1-DGlcNAc 

The antibacterial activity of 1-DGlcNAc was evaluated by the modified Oxford cup method [36]. *Xanthomonas* strains were cultivated in PSA medium (potato 300 g/L; Na_2_HPO_4_·12H_2_O 2 g/L; Ca(NO_3_)_2_·4H_2_O 0.5 g/L; tryptone, 5 g/L; and sucrose, 10 g/L) or on solid PSA medium added with 2% agar. Bacterial pathogens were cultured on Luria-Bertani medium (LB) plates.

1 mL of logarithmic phase bacterial culture was added to 50 mL of the medium, and 30 mL of this culture was shaken and poured into sterile Petri dishes (90 × 15 mm). After the solidification of the agar, the sterile stainless steel cylinders (6 × 10 mm) were placed on the surface of the agar and filled with 100 μL of the test samples at 100 μg/mL. Plates inoculated with *E. coli*, *S. aureus*, and *S. typhimurium* were incubated at 37 °C for 24 h. Plates with *P. solanacearum*, *P. syringae* and *Xanthomonas* strains were incubated at 28 °C for 24 h. The minimal inhibitory concentration (MIC) was determined by the cylinder plate method. The inhibitory zone diameter of 100 μL 1-DGlcNAc at 25, 50, 100, and 200 μg/mL was measured by Vernier caliper. Derived from the law of diffusion, the log value of the total concentration of antibiotics was linear to the square of the inhibitory zone diameter. The experiment was repeated three times. The MIC values were calculated using Microsoft Excel (version 2010 software, Microsoft Corporation, Redmond, WA, USA). Regression analyses were conducted using a linear regression model implemented in Excel. 

The assay for antifungal activity was executed on 90 mm × 15 mm Petri plates containing 30 mL of potato dextrose agar. After a mycelial colony had developed, sterile stainless steel cylinders (6 × 10 mm) were placed around and at a distance of 2 cm from the rim of the l colony and filled with 100 μL of tested samples at 100 μg/mL. Plates with *P. grisea*, and *S. sclerotiorum* were incubated at 20 °C for 72 h. The other plates were incubated at 28 °C for 72 h. The experiment was repeated three times.

### 3.8. Electron Microscopy Studies 

Scanning electron microscopy (SEM) and transmission electron microscopy (TEM) analyses were used to determine the effects of 1-DGlcNAc on Xanthomonas cells at the ultrastructural level. X. oryzae pv. oryzae PXO99A cells treated with 100 μg/mL 1-DGlcNAc were centrifuged and prefixed with 2.5% glutaraldehyde. Fixed cells were rinsed three times for 10 min each with 100 mM phosphate buffer, postfixed for 3 h in 1% osmium tetroxide, and dehydrated through an ethanol gradient. For SEM analysis, samples were coated with gold and analyzed on a Hitachi SU8010 scanning electron microscope (Hitachi, Tokyo, Japan). For TEM analysis, samples were applied to carbon-coated grids, sectioned with an ultramicrotome and examined under a FEI Tecani G20 TWIN transmission electron microscope.

### 3.9. Quantitative Real Time-PCR Analysis

qRT-PCR was assessed according to the method described by Wu et al. [37]. For the determination of gene expression, *X. oryzae* pv. *oryzae* PXO99A was exposed to 100 μg/mL 1-DGlcNAc in water for 3 h. Total RNA was extracted using a Bacterial RNA Kit (Vazyme, Nanjing, China) according to the manufacturer’s instructions. First-strand cDNA was synthesized using Reverse Transcriptase (Vazyme) with random hexamer primers and the resulting cDNA was used as the template for subsequent PCR amplification. qRT-PCR was performed with SYBR Green Master Mix (Vazyme) using a ViiA^TM7^ Real-Time PCR Detection System. Gene 16S rRNA was used as the internal reference for normalization. Primers for these genes are listed in Table S1.

### 3.10. PharmMapper Server

PharmMapper is a freely accessed web-server designed to identify potential target candidates for small molecules using the pharmacophore mapping approach [38,39]. The mol2 file of 1-DGlcNAc was uploaded. The druggable pharmacophore models (v2017, 16159) and pharmacophore models whose pKd ≥6.0 (v2017, 52431) were selected as targets sets respectively. The top 300 reserved matched targets were selected with default parameters.

## Figures and Tables

**Table 1:** ^1^H and ^13^C NMR signal assignments for 1-DGlcNAc and HMBC correlations (in MeOD).

No.	^1^H	Group	Multiplicity (*J*-Value)	^13^C	HMBC
1	3.13	C1H	t (11.0)	69.2	C2, C3, C5
	3.90	C1H’	dd (11.0, 5.2)	69.2	C2, C3, C4, C5
2	3.81	C2H	ddd(11.0, 10.1, 5.2)	53.2	C1, C3, C4, C7
3	3.41	C3H	dd (10.1, 8.6)	77.0	C1, C2, C4, C5
4	3.28	C4H	dd (9.7, 8.6)	72.6	C2, C3, C5, C6
5	3.19	C5H	ddd (9,7, 6.0, 2.2)	82.7	C1, C3, C4, C6
6	3.63	C6H	dd (11.9, 6.0)	63.2	C4, C5
	3.84	C6H’	dd (11.9, 2.2)	63.2	C4, C5
7	-	COCH3	-	174.2	-
8	1.97	COCH3	s	22.9	C1, C2, C3, C7

dd: doublet of doublets; ddd: doublet of doublets of doublets; s: singlet; t: triplet.

**Table 2:** Antibacterial spectrum assay.

Pathogenic Bacteria	Antibacterial Effect	
	1-DGlcNAc	2-Acetamido-1,5-anhydro-2-deoxy-d-mannitol
*Xanthomonas oryzae* pv. *oryzae* PXO99A	+	-
*Xanthomonas oryzae* pv. *oryzae* PXOΔ00069	+	-
*Xanthomonas campestris* pv. *campestris* str.8004	-	-
*Xanthomonas oryzae* pv. *oryzicola* RS105	-	-
*Escherichia coli*	-	-
*Staphylococcus aureus*	-	-
*Salmonella typhimurium*	-	-
*Pseudomonas solanacearum*	-	-
*Pseudomonas syringae*	-	-

+: active, -: no effect.

**Table 3:** Pharmacophore candidates of 1-DGlcNAc identified by pharmMapper.

Rank	PDB ID	Normalized Fit Score	Target Name
2	2C68	0.4792	cell division protein kinase 2
64	1V0P	0.3561	cell division control protein 2 homolog
235	1A4R	0.2595	cell division control protein 42 homolog
239	1RQ2	0.258	cell division protein ftsZ

## Supplementary Materials

The following are available online at http://www.mdpi.com/1660-3397/16/2/52/s1, Figure S1: 1D ^1^H spectrum of 1-DGlcNAc and signal; assignments, Figure S2: ^1^H–^1^H TOCSY 2D NMR spectra for 1-DGlcNAc and signal assignments, Figure S3: ^1^H–^1^H COSY 2D NMR spectra for 1-DGlcNAc and signal assignments, Figure S4: ^1^H–^1^H JRES 2D NMR spectra for 1-DGlcNAc and signal assignments, Figure S5: The ^1^H–^13^C HSQC NMR spectra for 1-DGlcNAc and signal assignments, Figure S6: The ^1^H–^13^C HMBC NMR spectra for 1-DGlcNAc and signal assignments, Figure S7: ^1^H NMR spectroscopy of isolated compound (a), chemically synthetic compound of 2-acetamido-1,5-anhydro-2-deoxy-d-mannitol (b), chemically synthetic compound of 2-acetamido-1,5-anhydro-2-deoxy-d-glucitol, Figure S8: Calculated and experimental ECD spectra of the isolated and synthesized compound. Table S1: DNA primers used in this study. Table S2: The top 300 pharmacophore candidates identified via pharmMapper.
