# Supplementary material for: Identification, Characteristics and Mechanism of 1-Deoxy-N-acetylglucosamine from Deep-Sea Virgibacillus dokdonensis MCCC 1A00493
Source: Mar Drugs. 2018 Feb 7;16(2):52. doi: 10.3390/md16020052 (PMC5852480; doi:10.3390/md16020052)
Supplement: Supplementary file 1 [file marinedrugs-16-00052-s001.pdf]

## Supplementary Materials

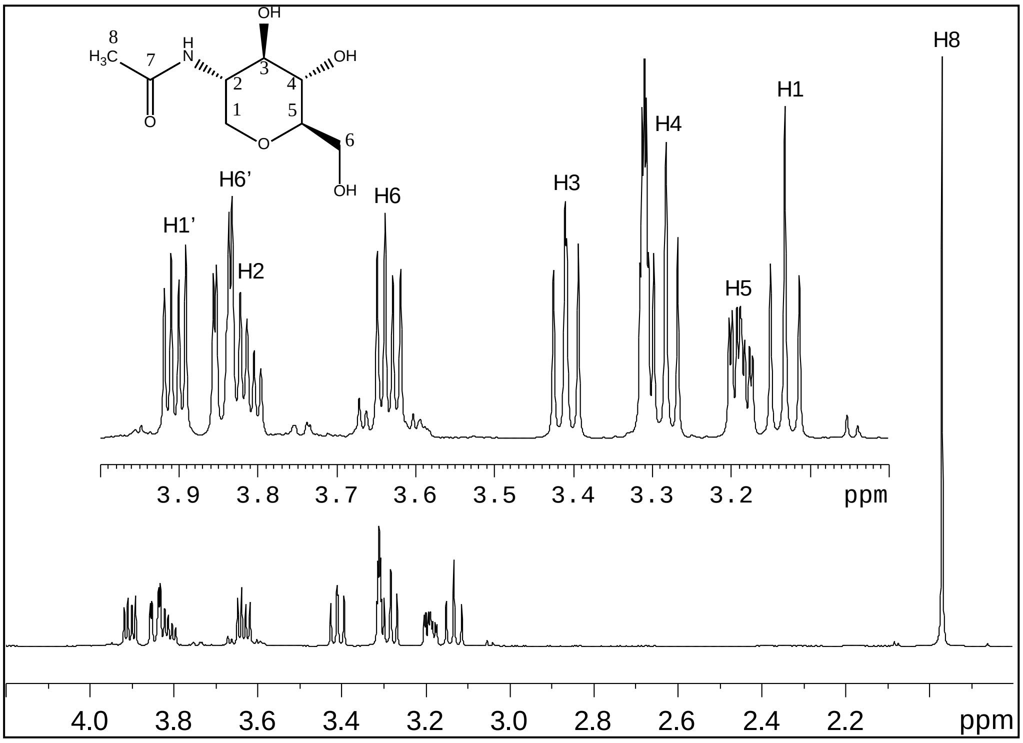


Figure S1. 1D $^{1}$H spectrum of 1-dglcnac and signal assignments

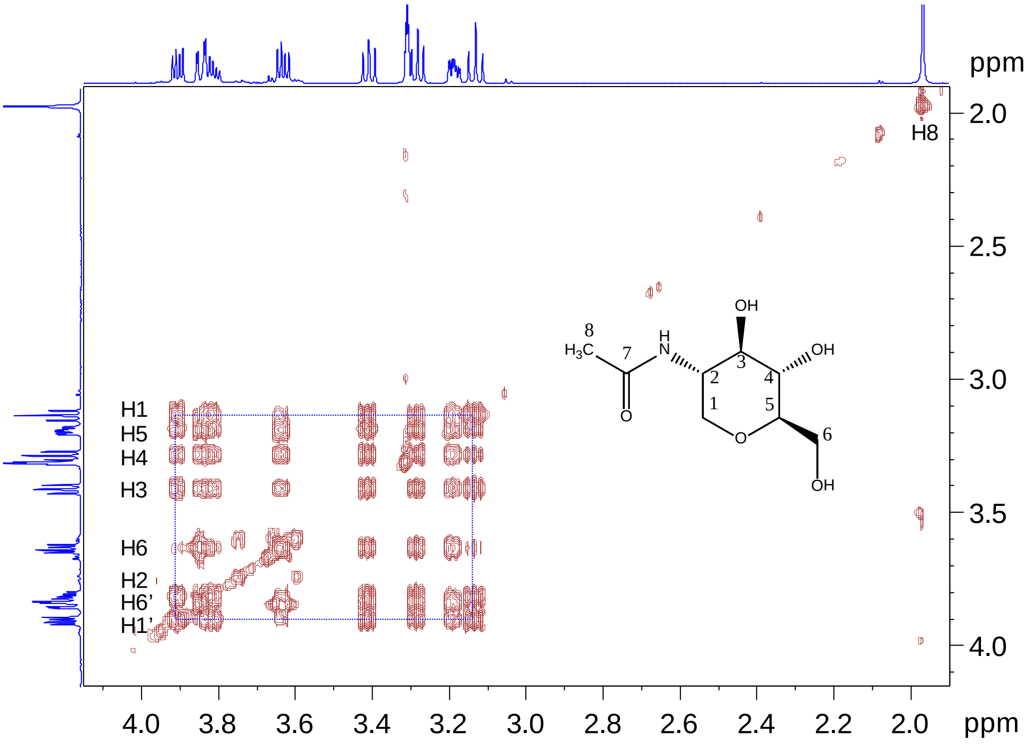


Figure S2. $^{1}$H-$^{1}$H TOCSY 2D NMR spectra for 1-dglcnac and signal assignments

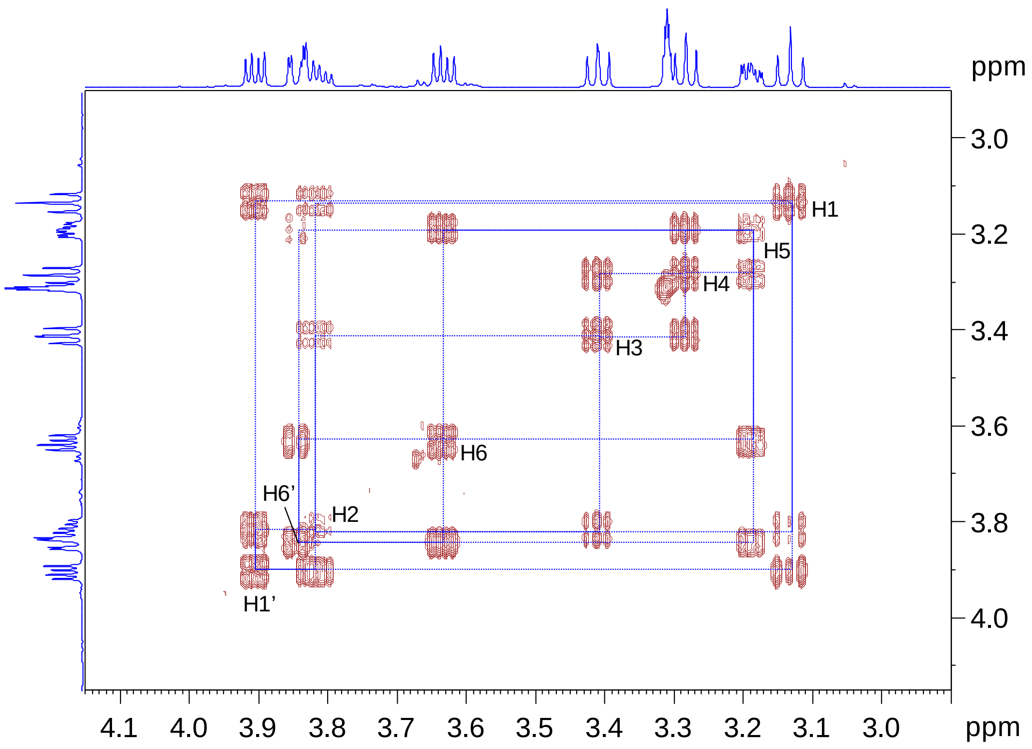


Figure S3. $^{1}H$-$^{1}H$ COSY 2D NMR spectra for 1-dglcnac and signal assignment

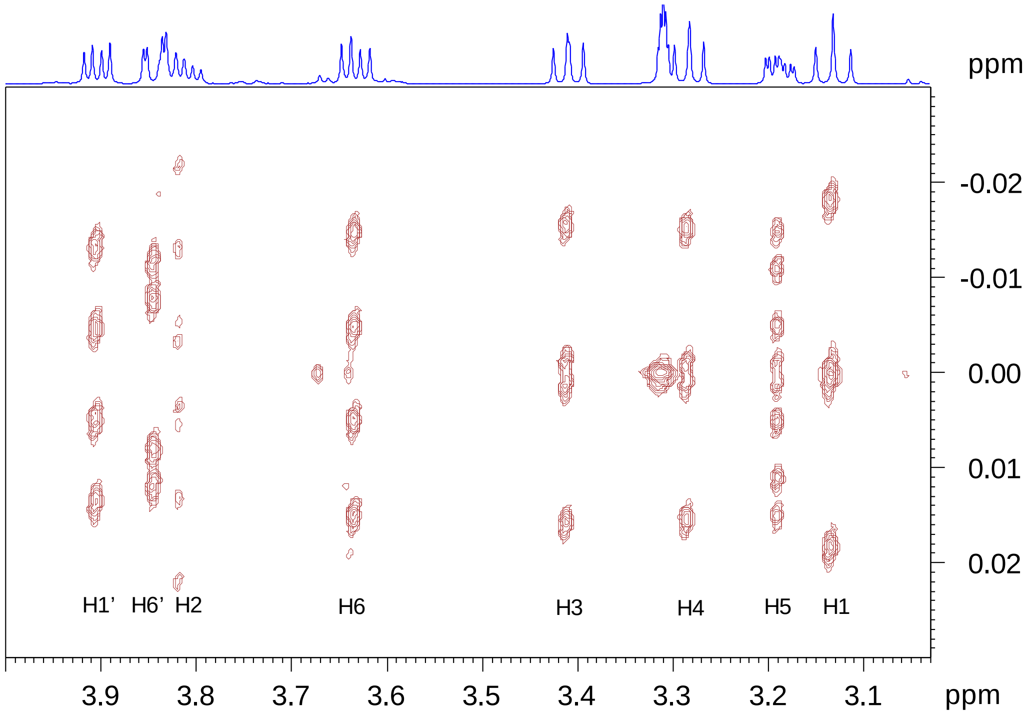


Figure S4. $^{1}H$-$^{1}H$ JRES 2D NMR spectra for 1-dglcnac and signal assignments

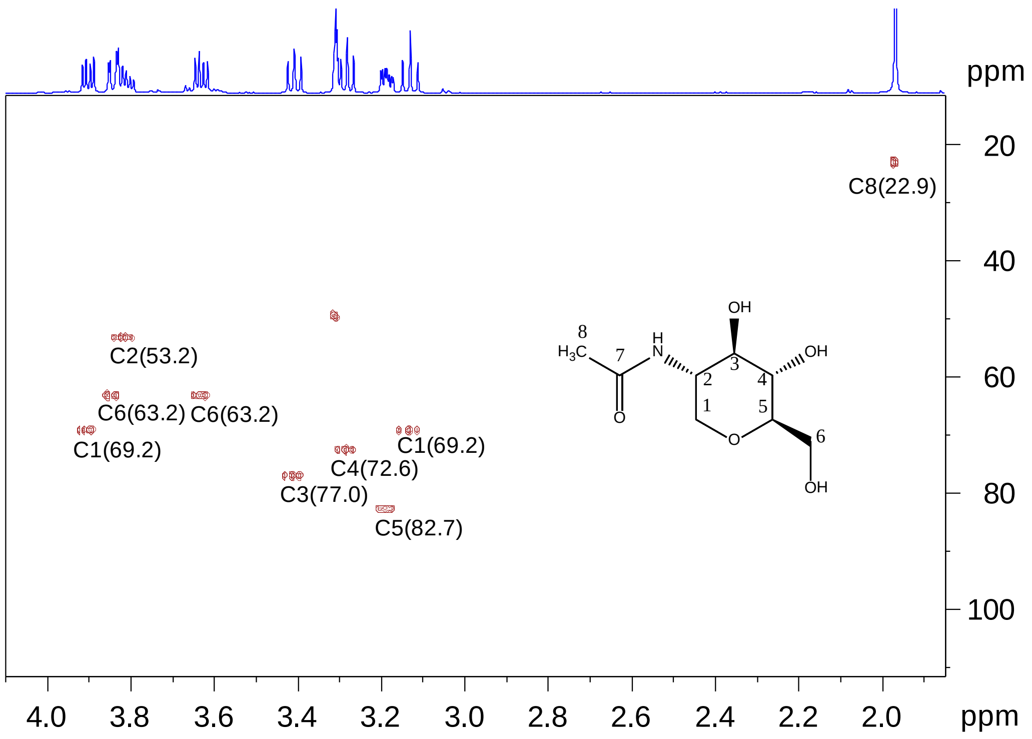


Figure S5. The $^{1}H$-$^{13}C$ HSQC NMR spectra for 1-dglcnac and signal assignments

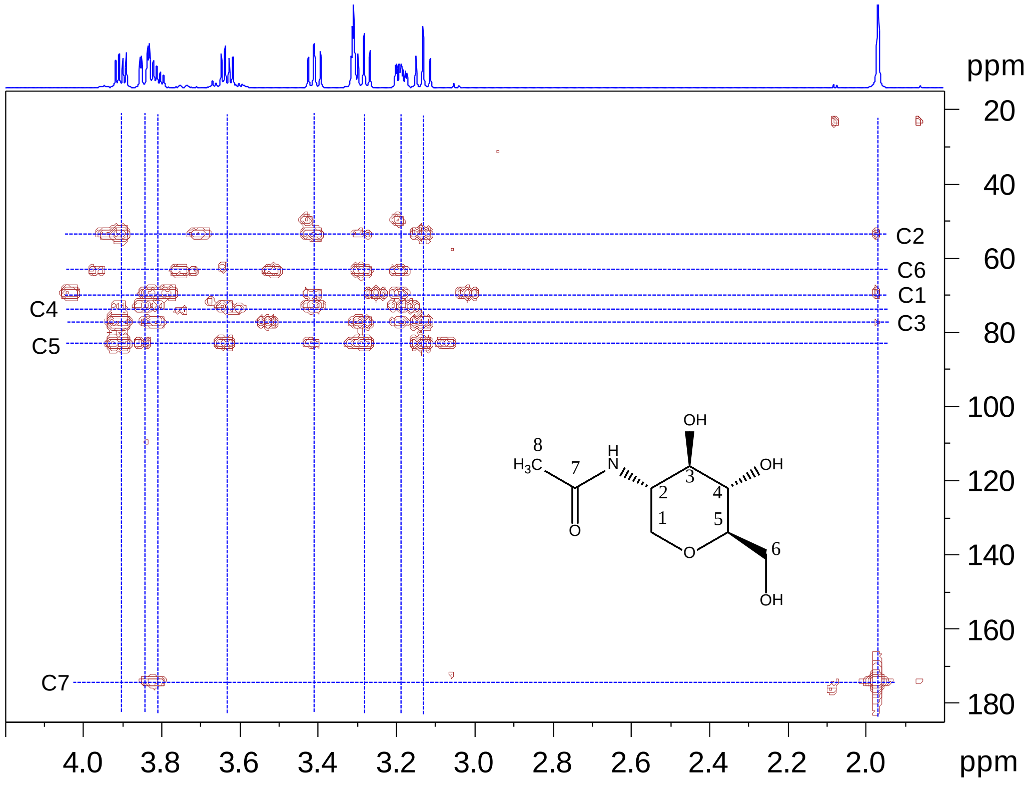


Figure S6. The $^{1}H$-$^{13}C$ HMBC NMR spectra for 1-dglcnac and signal assignments

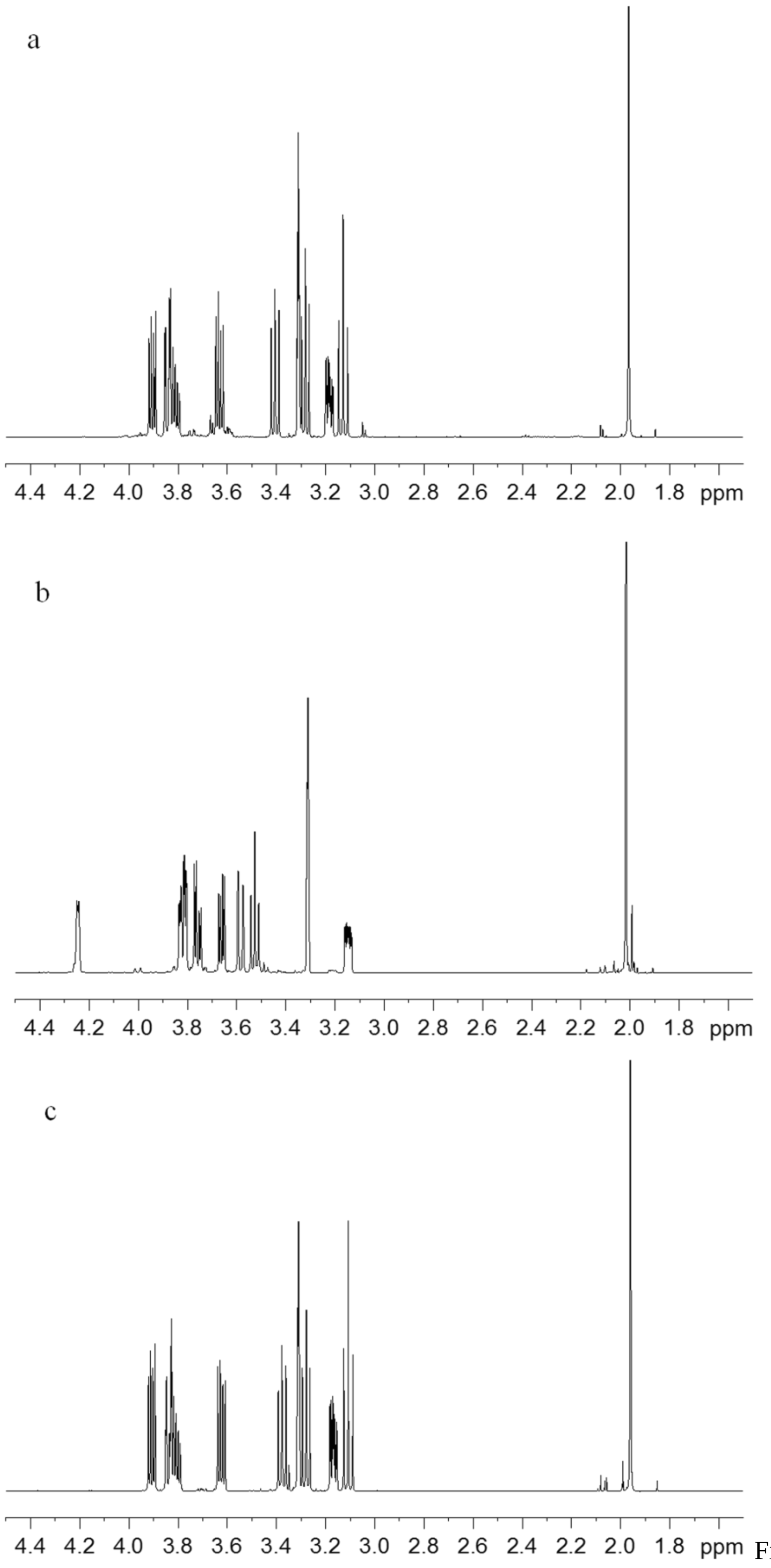


Figure S7. $^{1}$HNMR spectroscopy of isolated compound (a), chemically synthetic compound of 2-acetamido-1,5-anhydro-2-deoxy-D-mannitol (b), chemically synthetic compound of 2-acetamido-1,5-anhydro-2-deoxy-D-glucitol

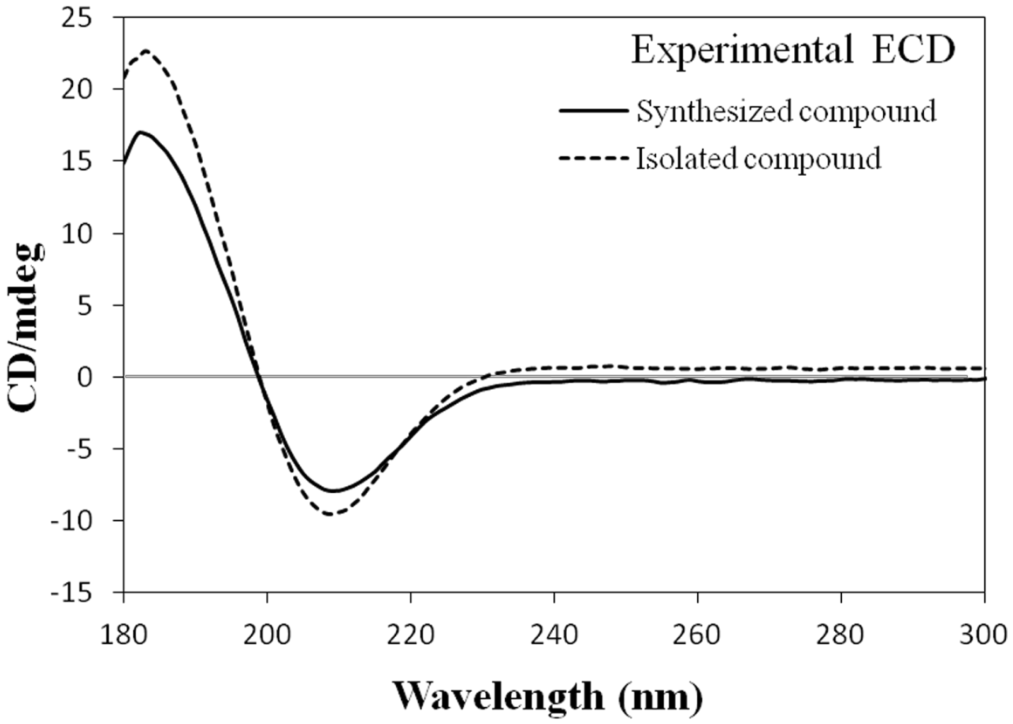


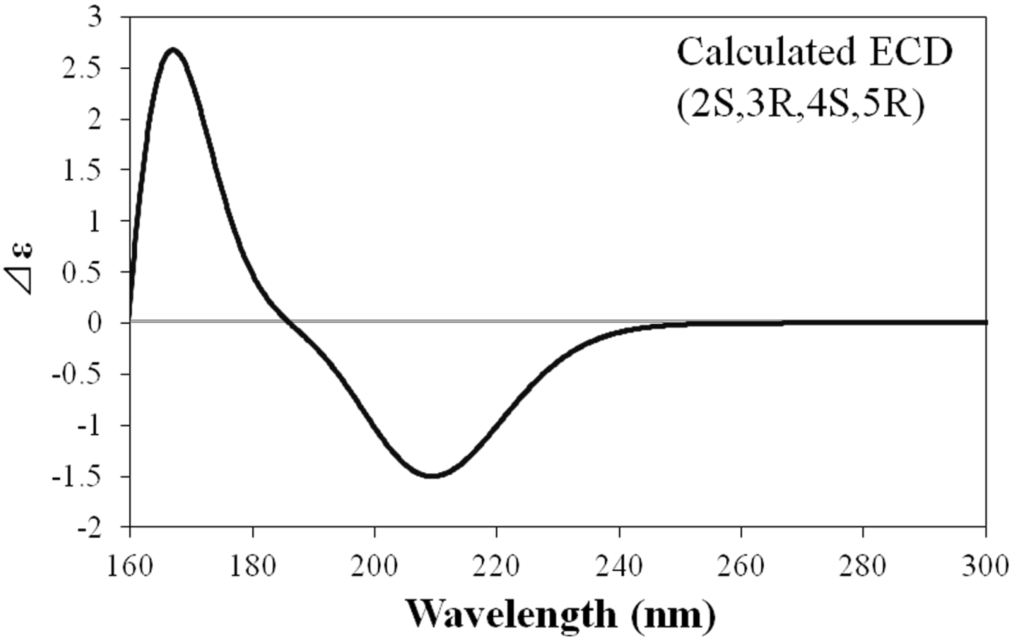


Figure S8. Calculated and experimental ECD spectra of the isolated and synthesized compound.

Table S1. DNA primers used in this study.

| Name | Sequence (5´ -3´) |
|---|---|
| 16S-F | TGGCAACTAAGGACAAGGG |

| | |
|---|---|
| 16S-R | AAGCGGTGGAGTATGTGG |
| rpfF-F | GTTGGTCTGATAGCGGGTGA |
| rpfF-R | TGAAGAACCGCAGCGTGAG |
| gumD-F | TTTCATAGTGCGTGTTGTGCT |
| gumD-R | AACGACGCACGCATCACG |
| ftsZ-F | CTGCTGGACGATGTGAACCTG |
| ftsZ-R | ATAATCGTTGGGCAGGTCAGC |
| glmS-F | CCCCAGCGATTTGGCGTAC |
| glmS-R | GCGTATGCGAACCCACAGC |

Table S2. The top 300 pharmacophore candidates identified via pharmMapper

| Rank | PDB ID | Target Name | Number of Feature | Fit Score | Normalized Fit Score |
|---|---|---|---|---|---|
| 1 | 1XIE | Xylose isomerase | 8 | 3.96 | 0.495 |
| 2 | 2C68 | Cell division protein kinase 2 | 6 | 2.875 | 0.4792 |
| 3 | 1UOU | Thymidine phosphorylase | 6 | 2.841 | 0.4736 |
| 4 | 1NB6 | Genome polyprotein | 6 | 2.84 | 0.4734 |
| 5 | 1UKG | NONE | 12 | 5.665 | 0.4721 |
| 6 | 1G0I | Inositol-1-monophosphatase | 6 | 2.828 | 0.4714 |
| 7 | 2AFU | Glutaminyl-peptide cyclotransferase | 8 | 3.752 | 0.469 |
| 8 | 2H15 | Carbonic anhydrase 2 | 6 | 2.813 | 0.4688 |
| 9 | 1DMJ | Nitric oxide synthase, endothelial | 6 | 2.78 | 0.4634 |

| | | | | | |
|---|---|---|---|---|---|
| 10 | 1O9W | F17a-G fimbrial adhesin | 12 | 5.527 | 0.4606 |
| 11 | 1LG1 | Chitotriosidase-1 | 6 | 2.763 | 0.4606 |
| 12 | 1TW5 | Beta-1,4-galactosyltransferase 1 | 10 | 4.582 | 0.4582 |
| 13 | 1TKU | 3,4-dihydroxy-2-butanone 4-phosphate synthase | 8 | 3.616 | 0.452 |
| 14 | 1TDB | Thymidylate synthase | 10 | 4.509 | 0.4509 |
| 15 | 6RSA | Ribonuclease pancreatic | 10 | 4.421 | 0.4421 |
| 16 | 1MRK | Ribosome-inactivating protein alpha-trichosanthin | 6 | 2.647 | 0.4411 |
| 17 | 1O70 | Fasciclin-1 | 8 | 3.524 | 0.4405 |
| 18 | 1QFX | 3-phytase B | 6 | 2.621 | 0.4368 |
| 19 | 1Q0N | 2-amino-4-hydroxy-6-hydroxymethyldihy dropteridine pyrophosphokinase | 8 | 3.441 | 0.4301 |
| 20 | 6CGT | Cyclomaltodextrin glucanotransferase | 10 | 4.261 | 0.4261 |
| 21 | 1T7Q | Carnitine O-acetyltransferase | 8 | 3.398 | 0.4248 |
| 22 | 1O7D | Lysosomal alpha-mannosidase | 10 | 4.215 | 0.4215 |
| 23 | 1FA2 | Beta-amylase | 8 | 3.361 | 0.4201 |
| 24 | 1UQX | Probable sugar-binding lectin protein | 14 | 5.841 | 0.4172 |
| 25 | 1JDN | Atrial natriuretic peptide clearance receptor | 6 | 2.493 | 0.4155 |
| 26 | 1UBY | Farnesyl pyrophosphate synthetase | 7 | 2.905 | 0.415 |
| 27 | 1MVN | Phosphopantothenoylcysteine decarboxylase | 6 | 2.438 | 0.4064 |
| 28 | 1O41 | Proto-oncogene tyrosine-protein kinase Src | 8 | 3.232 | 0.404 |
| 29 | 1DY4 | Exoglucanase 1 | 8 | 3.195 | 0.3994 |

| | | | | | |
|---|---|---|---|---|---|
| 30 | 1IMB | Inositol monophosphatase | 12 | 4.767 | 0.3972 |
| 31 | 1NLJ | Cathepsin K | 7 | 2.767 | 0.3953 |
| 32 | 1C7S | Chitobiase | 12 | 4.703 | 0.3919 |
| 33 | 1PR1 | Purine nucleoside phosphorylase deoD-type | 11 | 4.297 | 0.3906 |
| 34 | 1GIC | Concanavalin-A | 12 | 4.656 | 0.388 |
| 35 | 1PTG | 1-phosphatidylinositol phosphodiesterase | 12 | 4.594 | 0.3828 |
| 36 | 1GPJ | Glutamyl-tRNA reductase | 8 | 3.047 | 0.3809 |
| 37 | 1OX6 | Imidazole glycerol phosphate synthase hisHF | 7 | 2.647 | 0.3782 |
| 38 | 1MZE | Hypoxia-inducible factor 1-alpha inhibitor | 9 | 3.398 | 0.3776 |
| 39 | 1UNL | Cyclin-dependent kinase 5 activator 1 | 7 | 2.634 | 0.3762 |
| 40 | 1MSA | Mannose-specific lectin | 12 | 4.513 | 0.3761 |
| 41 | 1LT5 | Heat-labile enterotoxin B chain | 12 | 4.498 | 0.3748 |
| 42 | 1GOR | Endo-1,4-beta-xylanase | 10 | 3.738 | 0.3738 |
| 43 | 2I3I | Baculoviral IAP repeat-containing protein 7 | 8 | 2.982 | 0.3728 |
| 44 | 1LJN | Lysozyme C | 12 | 4.463 | 0.3719 |
| 45 | 1QCI | Antiviral protein I | 8 | 2.969 | 0.3712 |
| 46 | 1E70 | Myrosinase MA1 | 14 | 5.196 | 0.3711 |
| 47 | 2FOY | Carbonic anhydrase 1 | 10 | 3.704 | 0.3704 |
| 48 | 9LDB | L-lactate dehydrogenase A chain | 7 | 2.589 | 0.3699 |
| 49 | 1Q33 | ADP-ribose pyrophosphatase, mitochondrial | 16 | 5.914 | 0.3696 |
| 50 | 1FWY | Bifunctional protein glmU | 18 | 6.648 | 0.3693 |
| 51 | 1PHW | 2-dehydro-3-deoxyphosphooctonate aldolase | 8 | 2.95 | 0.3688 |

| | | | | | |
|---|---|---|---|---|---|
| 52 | 1S3F | Purine trans deoxyribosylase | 10 | 3.683 | 0.3683 |
| 53 | 1FYS | Guanyl-specific ribonuclease T1 | 10 | 3.646 | 0.3646 |
| 54 | 1UWZ | Cytidine deaminase | 10 | 3.632 | 0.3632 |
| 55 | 1KR6 | Thermolysin | 8 | 2.906 | 0.3632 |
| 56 | 1SQ5 | Pantothenate kinase | 10 | 3.618 | 0.3618 |
| 57 | 1MQ6 | Coagulation factor X | 8 | 2.891 | 0.3614 |
| 58 | 1CW7 | Isocitrate dehydrogenase [NADP] | 8 | 2.884 | 0.3605 |
| 59 | 1REF | Endo-1,4-beta-xylanase 2 | 10 | 3.597 | 0.3597 |
| 60 | 1D7U | 2,2-dialkylglycine decarboxylase | 8 | 2.866 | 0.3583 |
| 61 | 1L8L | Phosphoserine phosphatase | 8 | 2.864 | 0.358 |
| 62 | 1M7P | Triosephosphate isomerase | 6 | 2.148 | 0.3579 |
| 63 | 2ANG | Angiogenin | 8 | 2.859 | 0.3574 |
| 64 | 1V0P | Cell division control protein 2 homolog | 7 | 2.493 | 0.3561 |
| 65 | 1PFY | Methionyl-tRNA synthetase | 10 | 3.56 | 0.356 |
| 66 | 1OD8 | Endo-1,4-beta-xylanase A | 12 | 4.269 | 0.3558 |
| 67 | 2BKV | Glucosamine-6-phosphate deaminase 1 | 10 | 3.558 | 0.3558 |
| 68 | 1RFG | Purine nucleoside phosphorylase | 10 | 3.55 | 0.355 |
| 69 | 1JDZ | S-methyl-5-thioadenosine phosphorylase | 12 | 4.243 | 0.3536 |
| 70 | 1IJE | Elongation factor 1-alpha | 8 | 2.826 | 0.3533 |
| 71 | 1KEJ | DNA nucleotidylexotransferase | 10 | 3.508 | 0.3508 |

| | | | | | |
|---|---|---|---|---|---|
| 72 | 1L0G | Beta-lactamase | 8 | 2.797 | 0.3497 |
| 73 | 1FA9 | Glycogen phosphorylase, liver form | 14 | 4.864 | 0.3474 |
| 74 | 1XOG | Neuraminidase | 8 | 2.777 | 0.3472 |
| 75 | 1EQC | Glucan 1,3-beta-glucosidase | 12 | 4.163 | 0.3469 |
| 76 | 1TG2 | Phenylalanine-4-hydroxylase | 10 | 3.446 | 0.3446 |
| 77 | 1AHA | Ribosome-inactivating protein momordin I | 7 | 2.4 | 0.3428 |
| 78 | 1T9B | Acetolactate synthase catalytic subunit, mitochondrial | 8 | 2.731 | 0.3414 |
| 79 | 1M8E | Nitric oxide synthase, inducible | 6 | 2.049 | 0.3414 |
| 80 | 1OCQ | Endoglucanase 5A | 10 | 3.402 | 0.3402 |
| 81 | 1W55 | Bifunctional enzyme ispD/ispF | 8 | 2.721 | 0.3401 |
| 82 | 1KI3 | Thymidine kinase | 8 | 2.718 | 0.3398 |
| 83 | 1MQO | Beta-lactamase II | 11 | 3.685 | 0.335 |
| 84 | 1XL8 | Peroxisomal carnitine O-octanoyltransferase | 8 | 2.677 | 0.3347 |
| 85 | 1W7M | Kynurenine--oxoglutarate transaminase 1 | 8 | 2.656 | 0.332 |
| 86 | 1GVG | Clavaminate synthase 1 | 10 | 3.318 | 0.3318 |
| 87 | 1C1L | Congerin-1 | 12 | 3.975 | 0.3313 |
| 88 | 1O51 | UPF0166 protein TM_0021 | 8 | 2.642 | 0.3302 |
| 89 | 1HYB | Nicotinamide-nucleotide adenylyltransferase | 13 | 4.285 | 0.3296 |
| 90 | 1AX0 | Lectin | 10 | 3.291 | 0.3291 |
| 91 | 1M4G | Aminoglycoside 2-N-acetyltransferase | 12 | 3.946 | 0.3288 |
| 92 | 3HL5 | Baculoviral IAP repeat-containing protein 4 | 9 | 2.956 | 0.3284 |

| | | | | | |
|---|---|---|---|---|---|
| 93 | 1UA4 | ADP-dependent glucokinase | 20 | 6.532 | 0.3266 |
| 94 | 1VL1 | 6-phosphogluconolactonase | 8 | 2.611 | 0.3264 |
| 95 | 1J2Z | Acyl-[acyl-carrier-protein]--UDP-N-acetyl glucosamine O-acyltransferase | 10 | 3.259 | 0.3259 |
| 96 | 1MVQ | Mannose/glucose-specific lectin Cramoll | 12 | 3.906 | 0.3255 |
| 97 | 1W1A | Probable polysaccharide deacetylase pdaA | 10 | 3.255 | 0.3255 |
| 98 | 1LP6 | Orotidine 5-phosphate decarboxylase | 12 | 3.903 | 0.3253 |
| 99 | 1JS3 | Aromatic-L-amino-acid decarboxylase | 11 | 3.578 | 0.3253 |
| 100 | 1DCP | Pterin-4-alpha-carbinolamine dehydratase | 8 | 2.582 | 0.3227 |
| 101 | 1MD9 | 2,3-dihydroxybenzoate-AMP ligase | 12 | 3.852 | 0.321 |
| 102 | 1NP0 | Beta-hexosaminidase subunit beta | 8 | 2.567 | 0.3209 |
| 103 | 1T1S | 1-deoxy-D-xylulose 5-phosphate reductoisomerase | 8 | 2.557 | 0.3197 |
| 104 | 1HQL | GSI-B4 isolectin | 12 | 3.809 | 0.3174 |
| 105 | 5FIV | Pol polyprotein | 9 | 2.852 | 0.3169 |
| 106 | 3BM9 | Heat shock protein HSP 90-alpha | 8 | 2.535 | 0.3169 |
| 107 | 1JCJ | Deoxyribose-phosphate aldolase | 8 | 2.532 | 0.3164 |
| 108 | 1GQT | Ribokinase | 14 | 4.397 | 0.3141 |
| 109 | 1LIJ | Adenosine kinase | 14 | 4.394 | 0.3139 |
| 110 | 1H0R | 3-dehydroquinate dehydratase | 14 | 4.384 | 0.3131 |
| 111 | 3FZK | Heat shock cognate 71 kDa protein | 10 | 3.128 | 0.3128 |
| 112 | 4CTS | Citrate synthase, mitochondrial | 9 | 2.811 | 0.3123 |
| 113 | 1QK5 | Hypoxanthine-guanine-xanthine phosphoribosyltransferase | 12 | 3.744 | 0.312 |

| | | | | | |
|---|---|---|---|---|---|
| 114 | 1DC4 | Glyceraldehyde-3-phosphate dehydrogenase A | 12 | 3.74 | 0.3117 |
| 115 | 1DOG | Glucoamylase I | 12 | 3.737 | 0.3114 |
| 116 | 1GHV | Prothrombin | 7 | 2.175 | 0.3108 |
| 117 | 1ISS | Metabotropic glutamate receptor 1 | 9 | 2.796 | 0.3106 |
| 118 | 1MS6 | Cathepsin S | 9 | 2.775 | 0.3084 |
| 119 | 1HI3 | Non-secretory ribonuclease | 12 | 3.696 | 0.308 |
| 120 | 1UV5 | Glycogen synthase kinase-3 beta | 9 | 2.762 | 0.3069 |
| 121 | 1F0U | Cationic trypsin | 8 | 2.455 | 0.3068 |
| 122 | 1LTK | Phosphoglycerate kinase | 10 | 3.067 | 0.3067 |
| 123 | 1MDO | UDP-4-amino-4-deoxy-L-arabinose--oxoglutarate aminotransferase | 12 | 3.677 | 0.3065 |
| 124 | 1JC9 | Techylectin-5A | 8 | 2.45 | 0.3063 |
| 125 | 1PQP | Aspartate-semialdehyde dehydrogenase | 10 | 3.057 | 0.3057 |
| 126 | 1DYT | Eosinophil cationic protein | 8 | 2.443 | 0.3054 |
| 127 | 1MUO | Serine/threonine-protein kinase 6 | 7 | 2.125 | 0.3036 |
| 128 | 1KIZ | Trichodiene synthase | 9 | 2.728 | 0.3032 |
| 129 | 5ENL | Enolase 1 | 10 | 3.031 | 0.3031 |
| 130 | 1DAR | Elongation factor G | 12 | 3.636 | 0.303 |
| 131 | 3BIF | 6-phosphofructo-2-kinase/fructose-2,6-bisphosphatase 4 | 10 | 3.028 | 0.3028 |
| 132 | 1QF3 | Galactose-binding lectin | 10 | 3.016 | 0.3016 |
| 133 | 1A0G | D-alanine aminotransferase | 14 | 4.221 | 0.3015 |
| 134 | 1MOQ | Glucosamine--fructose-6-phosphate aminotransferase [isomerizing] | 14 | 4.218 | 0.3013 |
| 135 | 1GJ8 | Urokinase-type plasminogen activator | 12 | 3.601 | 0.3001 |

| | | | | | |
|---|---|---|---|---|---|
| 136 | 1UOW | Synaptotagmin-1 | 8 | 2.393 | 0.2992 |
| 137 | 1J1R | Antiviral protein S | 7 | 2.088 | 0.2983 |
| 138 | 1XL1 | Glycogen phosphorylase, muscle form | 14 | 4.163 | 0.2973 |
| 139 | 1AMR | Aspartate aminotransferase | 14 | 4.161 | 0.2972 |
| 140 | 1HV6 | Alginate lyase | 12 | 3.562 | 0.2969 |
| 141 | 1T31 | Chymase | 12 | 3.557 | 0.2964 |
| 142 | 1IH7 | DNA polymerase | 8 | 2.37 | 0.2963 |
| 143 | 3GSB | Glutamate-1-semialdehyde 2,1-aminomutase | 12 | 3.541 | 0.2951 |
| 144 | 1L1R | Adenine phosphoribosyltransferase | 7 | 2.064 | 0.2949 |
| 145 | 1XLW | Cholinesterase | 8 | 2.357 | 0.2947 |
| 146 | 1DSY | Protein kinase C alpha type | 10 | 2.933 | 0.2933 |
| 147 | 1TUY | Acetate kinase | 10 | 2.932 | 0.2932 |
| 148 | 2DC8 | Cathepsin B | 8 | 2.342 | 0.2928 |
| 149 | 1IW0 | Heme oxygenase | 10 | 2.926 | 0.2926 |
| 150 | 1OJO | Hyaluronate lyase | 8 | 2.34 | 0.2925 |
| 151 | 1QH5 | Hydroxyacylglutathione hydrolase, mitochondrial | 9 | 2.631 | 0.2923 |
| 152 | 1UC2 | UPF0027 protein PH1602 | 10 | 2.92 | 0.292 |
| 153 | 1JAK | Calcium-activated potassium channel subunit beta-2 | 16 | 4.656 | 0.291 |
| 154 | 1F7L | Holo-[acyl-carrier-protein] synthase | 12 | 3.491 | 0.2909 |
| 155 | 5MDH | Malate dehydrogenase, cytoplasmic | 8 | 2.317 | 0.2897 |
| 156 | 2FUS | Fumarate hydratase class II | 8 | 2.316 | 0.2895 |
| 157 | 1AUA | SEC14 cytosolic factor | 10 | 2.887 | 0.2887 |

| | | | | | |
|---|---|---|---|---|---|
| 158 | 1KSV | Ribosomal small subunit pseudouridine synthase A | 12 | 3.46 | 0.2883 |
| 159 | 1WOQ | Inorganic polyphosphate/ATP-glucomannokinase | 16 | 4.604 | 0.2877 |
| 160 | 1WDI | S-adenosylmethionin | 10 | 2.877 | 0.2877 |
| 161 | 4PFK | 6-phosphofructokinase | 20 | 5.712 | 0.2856 |
| 162 | 1BR5 | Ricin | 10 | 2.856 | 0.2856 |
| 163 | 1SIR | Glutaryl-CoA dehydrogenase, mitochondrial | 9 | 2.569 | 0.2855 |
| 164 | 1JXN | Anti-H(O) lectin 1 | 12 | 3.416 | 0.2847 |
| 165 | 1G8G | Sulfate adenylyltransferase | 12 | 3.413 | 0.2844 |
| 166 | 1NVA | Pentafunctional AROM polypeptide | 10 | 2.844 | 0.2844 |
| 167 | 1GP5 | Leucoanthocyanidin dioxygenase | 10 | 2.836 | 0.2836 |
| 168 | 1X7N | Glucose-6-phosphate isomerase | 16 | 4.528 | 0.283 |
| 169 | 1MN0 | Aldose 1-epimerase | 12 | 3.392 | 0.2827 |
| 170 | 1MRX | Gag-Pol polyprotein | 12 | 3.375 | 0.2812 |
| 171 | 2BVW | Exoglucanase-6A | 16 | 4.489 | 0.2806 |
| 172 | 1Q4N | Alpha-amylase 1 | 10 | 2.806 | 0.2806 |
| 173 | 1NNK | Glutamate receptor 2 | 10 | 2.8 | 0.28 |
| 174 | 2EEO | Aspartate 1-decarboxylase | 9 | 2.52 | 0.28 |
| 175 | 1CT5 | UPF0001 protein YBL036C | 12 | 3.358 | 0.2798 |
| 176 | 1HG1 | L-asparaginase | 10 | 2.794 | 0.2794 |
| 177 | 1IA7 | Cellulase Cel9-M | 8 | 2.233 | 0.2791 |
| 178 | 3BZU | Corticosteroid 11-beta-dehydrogenase isozyme 1 | 10 | 2.79 | 0.279 |

| | | | | | |
|---|---|---|---|---|---|
| 179 | 1AXB | Beta-lactamase TEM | 10 | 2.787 | 0.2787 |
| 180 | 2SIM | Sialidase | 14 | 3.896 | 0.2783 |
| 181 | 1QPC | Proto-oncogene tyrosine-protein kinase LCK | 10 | 2.779 | 0.2779 |
| 182 | 1V0H | Ascorbate peroxidase | 10 | 2.774 | 0.2774 |
| 183 | 1B8U | Malate dehydrogenase | 10 | 2.773 | 0.2773 |
| 184 | 1IUC | Fucose-specific lectin | 12 | 3.324 | 0.277 |
| 185 | 1SOO | Adenylosuccinate synthetase | 12 | 3.322 | 0.2768 |
| 186 | 1UJP | Tryptophan synthase alpha chain | 10 | 2.763 | 0.2763 |
| 187 | 1UYQ | Beta-glucosidase A | 12 | 3.312 | 0.276 |
| 188 | 1WDA | Protein-arginine deiminase type-4 | 10 | 2.755 | 0.2755 |
| 189 | 2ARC | Arabinose operon regulatory protein | 16 | 4.389 | 0.2743 |
| 190 | 1ITU | Dipeptidase 1 | 9 | 2.464 | 0.2738 |
| 191 | 1P62 | Deoxycytidine kinase | 14 | 3.832 | 0.2737 |
| 192 | 1B3O | Inosine-5-monophosphate dehydrogenase 2 | 13 | 3.557 | 0.2737 |
| 193 | 1MEN | Trifunctional purine biosynthetic protein adenosine-3 | 10 | 2.737 | 0.2737 |
| 194 | 1G6I | Endoplasmic reticulum mannosyl-oligosaccharide 1,2-alpha-mannosidase | 10 | 2.737 | 0.2737 |
| 195 | 1YMX | Beta-lactamase CTX-M-9a | 9 | 2.457 | 0.273 |
| 196 | 1JZQ | Isoleucyl-tRNA synthetase | 18 | 4.905 | 0.2725 |
| 197 | 1MC0 | cGMP-dependent 3,5-cyclic phosphodiesterase | 13 | 3.54 | 0.2723 |

| | | | | | |
|---|---|---|---|---|---|
| 198 | 1H78 | Anaerobic ribonucleoside-triphosphate reductase | 14 | 3.796 | 0.2712 |
| 199 | 1ATL | Zinc metalloproteinase atrolysin-D | 10 | 2.708 | 0.2708 |
| 200 | 1TU7 | Glutathione S-transferase 2 | 14 | 3.788 | 0.2706 |
| 201 | 1R1I | Neprilysin | 12 | 3.246 | 0.2705 |
| 202 | 1O26 | Thymidylate synthase thyX | 14 | 3.775 | 0.2696 |
| 203 | 2W6Z | Biotin carboxylase | 10 | 2.696 | 0.2696 |
| 204 | 1Y4S | Chaperone protein htpG | 14 | 3.768 | 0.2692 |
| 205 | 1KZL | Riboflavin synthase alpha chain | 18 | 4.839 | 0.2688 |
| 206 | 1N97 | Cytochrome P450 | 12 | 3.214 | 0.2679 |
| 207 | 1CZA | Hexokinase-1 | 20 | 5.349 | 0.2674 |
| 208 | 2CHB | Cholera enterotoxin subunit B | 12 | 3.205 | 0.2671 |
| 209 | 1T4T | Arginase-1 | 11 | 2.932 | 0.2665 |
| 210 | 1JKI | Inositol-3-phosphate synthase | 14 | 3.722 | 0.2659 |
| 211 | 1B02 | Thymidylate synthase A | 16 | 4.252 | 0.2657 |
| 212 | 1FJ6 | Fructose-1,6-bisphosphatase 1 | 14 | 3.709 | 0.265 |
| 213 | 1RY2 | Acetyl-coenzyme A synthetase 1 | 14 | 3.705 | 0.2646 |
| 214 | 1KSU | Fumarate reductase flavoprotein subunit | 10 | 2.646 | 0.2646 |
| 215 | 1RZY | Histidine triad nucleotide-binding protein 1 | 17 | 4.489 | 0.2641 |
| 216 | 1IYP | Beta-lactamase Toho-1 | 13 | 3.43 | 0.2638 |
| 217 | 2AIG | Zinc metalloproteinase adamalysin-2 | 14 | 3.692 | 0.2637 |

| | | | | | |
|---|---|---|---|---|---|
| 218 | 1E8U | Hemagglutinin-neuraminidase | 17 | 4.476 | 0.2633 |
| 219 | 1TDJ | Threonine dehydratase biosynthetic | 10 | 2.628 | 0.2628 |
| 220 | 1PMQ | Mitogen-activated protein kinase 10 | 14 | 3.676 | 0.2626 |
| 221 | 1OON | Oxygen-insensitive NAD(P)H nitroreductase | 14 | 3.671 | 0.2622 |
| 222 | 1UWS | Beta-galactosidase | 14 | 3.671 | 0.2622 |
| 223 | 1OF8 | Phospho-2-dehydro-3-deoxyheptonate aldolase, tyrosine-inhibited | 10 | 2.622 | 0.2622 |
| 224 | 2SFP | Alanine racemase | 14 | 3.67 | 0.2621 |
| 225 | 1IPC | Eukaryotic translation initiation factor 4E | 10 | 2.619 | 0.2619 |
| 226 | 1HS6 | Leukotriene A-4 hydrolase | 14 | 3.664 | 0.2617 |
| 227 | 1NXJ | Regulator of ribonuclease activity A | 10 | 2.617 | 0.2617 |
| 228 | 1R0Y | Cystic fibrosis transmembrane conductance regulator | 17 | 4.448 | 0.2616 |
| 229 | 1MWM | Plasmid segregation protein parM | 12 | 3.136 | 0.2614 |
| 230 | 1QJI | Astacin | 14 | 3.658 | 0.2613 |
| 231 | 1MQQ | Alpha-glucuronidase | 18 | 4.695 | 0.2608 |
| 232 | 1RDW | Actin, alpha skeletal muscle | 10 | 2.607 | 0.2607 |
| 233 | 1KT8 | Branched-chain-amino-acid aminotransferase, mitochondrial | 14 | 3.645 | 0.2604 |
| 234 | 1XBX | 3-keto-L-gulonate-6-phosphate decarboxylase ulaD | 14 | 3.634 | 0.2595 |
| 235 | 1A4R | Cell division control protein 42 homolog | 10 | 2.595 | 0.2595 |
| 236 | 1A05 | 3-isopropylmalate dehydrogenase | 13 | 3.371 | 0.2593 |
| 237 | 1UN7 | N-acetylglucosamine-6-phosphate deacetylase | 18 | 4.665 | 0.2592 |

| | | | | | |
|---|---|---|---|---|---|
| 238 | 1A25 | Protein kinase C beta type | 8 | 2.067 | 0.2584 |
| 239 | 1RQ2 | Cell division protein ftsZ | 10 | 2.58 | 0.258 |
| 240 | 1JAQ | Neutrophil collagenase | 14 | 3.611 | 0.2579 |
| 241 | 2B4Y | NAD-dependent deacetylase sirtuin-5 | 14 | 3.606 | 0.2576 |
| 242 | 1MF0 | Adenylosuccinate synthetase isozyme 1 | 10 | 2.576 | 0.2576 |
| 243 | 1UEJ | Uridine-cytidine kinase 2 | 17 | 4.368 | 0.257 |
| 244 | 1GEX | Histidinol-phosphate aminotransferase | 12 | 3.083 | 0.2569 |
| 245 | 1SQS | Hypothetical protein | 12 | 3.081 | 0.2568 |
| 246 | 1E99 | Thymidylate kinase | 9 | 2.305 | 0.2561 |
| 247 | 1S3G | Adenylate kinase | 8 | 2.045 | 0.2557 |
| 248 | 1BKJ | NADPH-flavin oxidoreductase | 16 | 4.086 | 0.2554 |
| 249 | 1CKN | mRNA-capping enzyme | 11 | 2.808 | 0.2553 |
| 250 | 2V3D | Glucosylceramidase | 14 | 3.573 | 0.2552 |
| 251 | 2C6C | Glutamate carboxypeptidase 2 | 14 | 3.568 | 0.2548 |
| 252 | 1X92 | Phosphoheptose isomerase | 14 | 3.561 | 0.2543 |
| 253 | 1KRF | Mannosyl-oligosaccharide alpha-1,2-mannosidase | 14 | 3.554 | 0.2539 |
| 254 | 1B57 | Fructose-bisphosphate aldolase class 2 | 14 | 3.549 | 0.2535 |
| 255 | 1H4G | Glycoside hydrolase | 16 | 4.051 | 0.2532 |
| 256 | 1BB7 | Lysozyme C II | 16 | 4.034 | 0.2522 |
| 257 | 1RQJ | Geranyltranstransferase | 12 | 3.027 | 0.2522 |
| 258 | 1PS3 | Alpha-mannosidase 2 | 16 | 4.021 | 0.2513 |

| | | | | | |
|---|---|---|---|---|---|
| 259 | 1KC3 | dTDP-4-dehydrorhamnose reductase | 11 | 2.762 | 0.2511 |
| 260 | 1VRP | Creatine kinase M-type | 14 | 3.512 | 0.2508 |
| 261 | 1J01 | Exoglucanase/xylanase [Includes: Exoglucanase | 18 | 4.511 | 0.2506 |
| 262 | 1AB8 | Adenylate cyclase type 2 | 10 | 2.501 | 0.2501 |
| 263 | 2QCF | Uridine 5-monophosphate synthase | 18 | 4.495 | 0.2497 |
| 264 | 1YBU | PROBABLE LIGNIN PEROXIDASE LIPJ | 12 | 2.992 | 0.2494 |
| 265 | 1A6W | Ig heavy chain V region B1-8/186-2 | 11 | 2.741 | 0.2492 |
| 266 | 2NCD | Protein claret segregational | 14 | 3.487 | 0.249 |
| 267 | 1KF0 | Phosphoglycerate kinase 1 | 13 | 3.237 | 0.249 |
| 268 | 1R5H | Methionine aminopeptidase 2 | 15 | 3.733 | 0.2489 |
| 269 | 1NW6 | Modification methylase RsrI | 14 | 3.479 | 0.2485 |
| 270 | 1IYH | Glutathione-requiring prostaglandin D synthase | 11 | 2.725 | 0.2477 |
| 271 | 2GG0 | Methionine aminopeptidase | 16 | 3.961 | 0.2476 |
| 272 | 1PZ2 | Alpha-N-arabinofuranosidase | 14 | 3.465 | 0.2475 |
| 273 | 1YLZ | Extended-spectrum beta-lactamase CTX-M-14 | 11 | 2.721 | 0.2473 |
| 274 | 1MIY | CCA-adding enzyme | 14 | 3.461 | 0.2472 |
| 275 | 1X6V | Bifunctional 3-phosphoadenosine 5-phosphosulfate synthetase 1 | 14 | 3.46 | 0.2471 |
| 276 | 2JFF | UDP-N-acetylmuramoylalanine--D-glutamate ligase | 11 | 2.715 | 0.2469 |
| 277 | 1SMO | Triggering receptor expressed on myeloid cells 1 | 14 | 3.451 | 0.2465 |
| 278 | 1PCM | Phosphomannomutase/phosphoglucomutase | 18 | 4.429 | 0.2461 |

| | | | | | |
|---|---|---|---|---|---|
| 279 | 1SDE | D-alanyl-D-alanine carboxypeptidase | 11 | 2.703 | 0.2458 |
| 280 | 1M9A | Glutathione S-transferase class-mu 26 kDa isozyme | 11 | 2.699 | 0.2454 |
| 281 | 1P9P | tRNA | 18 | 4.413 | 0.2452 |
| 282 | 1KDT | Cytidylate kinase | 10 | 2.447 | 0.2447 |
| 283 | 1J53 | DNA polymerase III subunit epsilon | 15 | 3.669 | 0.2446 |
| 284 | 1QJQ | Ferrichrome-iron receptor | 15 | 3.668 | 0.2445 |
| 285 | 6FIT | Bis(5-adenosyl)-triphosphatase | 10 | 2.439 | 0.2439 |
| 286 | 1NC3 | 5-methylthioadenosine/S-adenosylhomocy steine nucleosidase | 16 | 3.9 | 0.2438 |
| 287 | 1M0U | Glutathione S-transferase S1 | 11 | 2.679 | 0.2436 |
| 288 | 1P6K | Nitric oxide synthase, brain | 10 | 2.435 | 0.2435 |
| 289 | 1AQJ | Modification methylase TaqI | 14 | 3.404 | 0.2432 |
| 290 | 1V4S | Glucokinase | 10 | 2.431 | 0.2431 |
| 291 | 1MXI | Probable tRNA/rRNA methyltransferase HI0766 | 16 | 3.875 | 0.2422 |
| 292 | 1UB3 | Lyase | 14 | 3.389 | 0.2421 |
| 293 | 1U27 | Cytohesin-2 | 20 | 4.824 | 0.2412 |
| 294 | 1BUC | Acyl-CoA dehydrogenase, short-chain specific | 12 | 2.89 | 0.2408 |
| 295 | 1MC3 | Glucose-1-phosphate thymidylyltransferase 2 | 20 | 4.815 | 0.2407 |
| 296 | 1PPV | Isopentenyl-diphosphate Delta-isomerase | 10 | 2.402 | 0.2402 |
| 297 | 1NZC | DTDP-4-dehydrorhamnose 3,5-epimerase | 14 | 3.362 | 0.2401 |
| 298 | 1J0A | Putative 1-aminocyclopropane-1-carboxylate deaminase | 16 | 3.835 | 0.2397 |
| 299 | 1EYE | Dihydropteroate synthase 1 | 14 | 3.356 | 0.2397 |
